# Individual size variation reduces spatial variation in abundance of tree community assemblage, not of tree populations

**DOI:** 10.1002/ece3.3594

**Published:** 2017-11-09

**Authors:** Hua‐Feng Wang, Meng Xu

**Affiliations:** ^1^ Hainan Key Laboratory for Sustainable Utilization of Tropical Bioresources Institute of Tropical Agriculture and Forestry Hainan University Haikou China; ^2^ Department of Mathematics Pace University New York NY USA

**Keywords:** aboveground biomass, Diaoluo Mountain, individual size variation, plant community, spatial variation, taxonomy

## Abstract

Research on individual trait variation has gained much attention because of its implication for ecosystem functions and community ecology. The effect of individual variation on population and community abundance (number of individuals) variation remains scarcely tested. Using two established ecological scaling laws (Taylor's law and abundance–size relationship), we derived a new scaling relationship between the individual size variation and spatial variation of abundance. Tested against multi‐plot tree data from Diaoluo Mountain tropical forest in Hainan, China, the new scaling relationship showed that individual size variation reduced the spatial variation of community assemblage abundance, but not of taxon‐specific population abundance. The different responses of community and population to individual variation were reflected by the validity of the abundance–size relationship. We tested and confirmed this scaling framework using two measures of individual tree size: aboveground biomass and diameter at breast height. Using delta method and height‐diameter allometry, we derived the analytic relation of scaling exponents estimated under different individual size measures. In addition, we used multiple regression models to analyze the effect of taxon richness on the relationship between individual size variation and spatial variation of population or community abundance, for taxon‐specific and taxon‐mixed data, respectively. This work offers empirical evidence and a scaling framework for the negative effect of individual trait variation on spatial variation of plant community. It has implications for forest ecosystem and management where the role of individual variation in regulating population or community spatial variation is important but understudied.

## 1 INTRODUCTION

Ecological research concerns the functioning and interaction of characteristics among individuals, populations, and communities. In particular, population ecology and community ecology emphasize species and their differences and downplay the role of individual variations. On the other hand, individual trait variation has been advocated as a key variable in regulating population dynamics and ecosystem functions (Bjørnstad & Hansen, 1994; Bolnick et al., 2011; Dochtermann & Gienger, 2012). Forsman and Wennersten (2016) reviewed numerous field and laboratory studies reporting that individual genetic or phenotypic variation enhanced ecological performances of species populations. Specifically, they cited five works documenting how individual genetic variation could reduce population fluctuation. In all works mentioned above, individual variation was used to infer temporal stability of species abundance. The effect of individual variation on the spatial variation of population and community abundance is understudied. On the other hand, research about the influence of individual variation on spatial variation of abundance often blurs the boundary between population and community.

Our goal here is to examine the relation between individual size variation and spatial variation of abundance of taxon‐specific populations and taxon‐mixed community. Spatial variation of abundance reflects species adaptation to heterogeneous habitats, resource acquisition, and competition that are relevant to conservation and human health, such as in species invasion (Hansen et al., 2013; Latzka, Hansen, Kornis, & Vander Zanden, 2016) and host‐parasite systems (Morand & Krasnov, 2008). Incorporating individual variation adds a new dimension to the studies of underlying mechanisms of spatial variation of abundance. For example, in tick‐borne diseases, impact of deer distribution on spatial variation of tick population is one of the key areas in Lyme disease research (Kilpatrick et al., 2017). Analysis of variations in the diet preference, parasite resistance, body size, and other traits among deer individuals can shed light on the understanding of spatial distribution of ticks, so that effective control plan can be designed.

Despite recognition of the influence of individual size variation on abundance variation, a theoretical framework that can account for their relation is still lacking. Moreover, how individual variation affects the spatial variation of community‐level and population‐level abundance remains largely untested empirically. In this work, we used two widely tested ecological scaling laws (i.e., Taylor's law and abundance–size relationship) to derive analytically a new scaling relationship relating the variance of individual size to spatial variance of population or community abundance (Xu, 2016). We then tested empirically these existing and new scaling patterns using taxon‐specific and taxon‐mixed tree data separately from the Diaoluo Mountain tropical forest in Hainan, China. Our results are as follows: (i) Spatial variation of assemblage abundance was a negative power‐law function of the individual size variation for tree community, as confirmed by the new scaling relationship; (ii) Individual size variation and spatial variation of abundance were not significantly correlated for taxon‐specific populations; (iii) Power exponent of the new scaling relationship can be predicted analytically using the parameter estimates of Taylor's law and abundance–size relationship for community‐level data; and (iv) Allometric theory provided analytic insight into the relation of scaling parameters under different individual size measures. Based on our findings, we speculated that taxonomic variation in resource acquisition and intertaxonomic competition explained the observed discrepancy in the effects of individual size variation on spatial variation of abundance between population and community.

The analytic derivation of the scaling framework used here was done in Xu (2016). We reviewed briefly Taylor's law and abundance–size relationship as they were the building blocks of our theory. We gave historical background and ecological interpretations of these existing scaling patterns. Moreover, we elaborated on how common variables shared by these patterns allowed their integration, which was used to derive the relationship between individual size variation and spatial variation of abundance at population and community levels.

Taylor's law states that the variance of population abundance of a single or a group of species is a power function of the mean population abundance (Taylor, 1961):

(1) $$\text{variance of abundance}=a{\left(\text{mean abundance}\right)}^{b},\;a>0.$$

Equation (1) (or its log‐linear form) has been confirmed for thousands of biological taxa (Eisler, Bartos, & Kertész, 2008). The power exponent *b* of Taylor's law was believed to contain species‐specific information about how population aggregates in space, with larger *b* indicating higher degree of aggregation. Despite numerous dynamic and spatial models have been proposed to explain Taylor's law (Anderson, Gordon, Crawley, & Hassell, 1982; Ballantyne, 2005; Cohen & Saitoh, 2016; Kilpatrick & Ives, 2003; Shi, Sandhu, & Reddy, 2016), a unified theory that can account for its presence in various ecological systems is still lacking. Recently developed statistical models reproduced successfully the mean–variance scaling relationship (Equation (1)), but failed to explain the specific value of *b* under biologically realistic conditions (Cohen & Xu, 2015; Xiao, Locey, & White, 2015). In Xu (2016) and the current work, we applied Taylor's law to individual size and hypothesized

(2) $$\text{variance of individual size}=c{\left(\text{mean individual size}\right)}^{d},\;c>0.$$

We called Equations (1) and (2) as the Taylor's law for abundance and Taylor's law for individual size, respectively. We tested both equations using taxon‐specific and taxon‐mixed data separately. We further tested Equation (2) with aboveground biomass (AGB) and diameter at breast height (dbh) as the individual size measure separately.

Abundance–size relationship manifests in many different forms, with two particular forms (local size–density relationship and cross‐community scaling relationship, see White, Ernest, Kerkhoff, & Enquist, 2007) testable for individuals in a single community. Specifically, local size–density relationship links average body size of a species to its population abundance. It often exhibited weak or triangular patterns due to taxonomic differences in resource acquisition (Brown & Maurer, 1987) or limited body size variation within single taxon (Currie, 1993). On the other hand, cross‐community scaling relationship describes the assemblage abundance of an entire community as a function of the average body size of all individuals within the community. The commonly found power‐law form of cross‐community scaling relationship reflects the energy partitioning among individuals of various sizes. A power‐law exponent of minus one indicated energy equivalence within the community (Long & Morin, 2005); however, such observation was not universal (Isaac, Storch, & Carbone, 2011). Here, we tested both forms of the abundance–size relationship using AGB and dbh as size measure separately. It is worth noting that, in our empirical analysis at the site scale, we used mean abundance per plot within a site (for taxon‐specific and taxon‐mixed abundance separately) and assumed the abundance–size relationship

(3) $$\text{mean abundance}=\alpha {\left(\text{mean individual size}\right)}^{\beta },\alpha >0,\beta <0.$$

Abundance–size relationship (Equation (3)) linked the explanatory variables in Taylor's law for abundance (“mean abundance” in Equation (1)) and Taylor's law for individual size (“mean individual size” in Equation (2)). Based on this observation and simple algebra, we related the response variables in Equations (1) and (2) as (see equation 7 in Xu, 2016)

(4) $$\begin{array}{cc} \text{variance of abundance} & =\;\gamma {\left(\text{variance of individual size}\right)}^{\eta }\\ & =\frac{a\alpha^{b}}{c^{\frac{\beta b}{d}}}{\left(\text{variance of individual size}\right)}^{\frac{\beta b}{d}}. \end{array}$$

We called Equation (4) the abundance–size variance relationship. The derived power‐law functional form and the negative exponent (β*b*/*d *<* *0 as β < 0, *b *>* *0, and *d *>* *0) (last term in Equation (4)) indicated that variance of individual size and spatial variance of abundance are negatively correlated. Our analysis tested the power‐law form of the abundance–size variance relationship and compared its power exponent estimated from data and predicted from Equations (1), (2), (3) (=β*b*/*d*). We repeated this analysis for taxon‐specific and taxon‐mixed data under different individual size measures (AGB and dbh) separately.

## 2 MATERIALS AND METHODS

### 2.1 Study site and data

**Figure 1:**
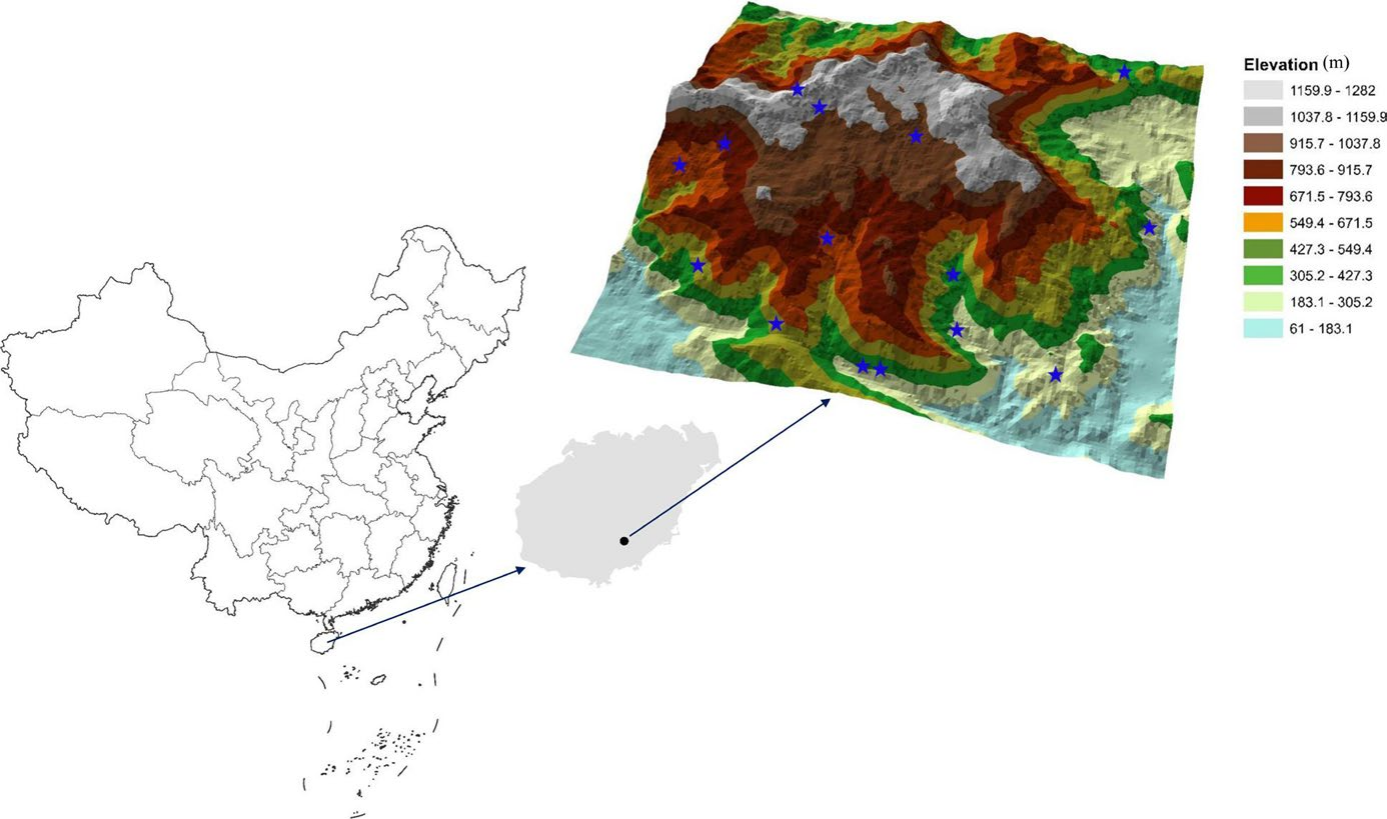
Geographic locations of 15 sampling sites (blue stars) of Diaoluo Mountain in Hainan, China

Data used for analysis were collected from the Diaoluoshan (Diaoluo Mountain) tropical forest (18.75°N, 109.87°E), located in the southeast of the Hainan province, China (Figure 1, Table 1). The region resides in a tropical maritime monsoon climate zone, with rainy season from May to October and dry season from November to April. The forest soil types are mainly moist, acidic, and mountain yellow. Its annual average temperature is 24.4°C with an average annual rainfall of 2,180.9 millimeters (mm). Diaoluo Mountain covers large areas of primary evergreen forests and secondary forests. The secondary forests were mainly recovered from the overlumbered areas in the 1950s. The average height of the plant community is 10 meters (m) with flat crown.

**Table 1:** Locations and characteristics of 15 sampling sites in the Diaoluo mountain tropical forest, Hainan province, China

Code	Location	Latitude	Longitude	Altitude (m)	Slope (%)	Aspect (°)
1	Dali Ridge	18.770	109.936	475	19.816	39.920
2	Xiaomei Reservoir	18.723	109.947	250	16.984	103.074
3	Back mountain of Beurea of retired staff	18.679	109.931	245	9.297	190.923
4	Shuixin	18.685	109.910	270	7.715	92.386
5	Shuixin Hydropower Station	18.698	109.906	395	16.403	85.054
6	Nanxi Station Citrus reticulata forests	18.672	109.896	255	22.788	186.553
7	Opposite forest of Southxi Station	18.672	109.893	265	23.708	172.807
8	Baishuikeng pit	18.675	109.873	515	19.368	228.252
9	Five kilometer far away from Baishuikeng pit	18.675	109.873	555	23.605	250.980
10	Baishui Pond	18.711	109.838	640	11.499	223.877
11	Baishui primary Forest	18.719	109.847	750	8.566	218.157
12	Dousi Bridge	18.697	109.878	665	14.679	75.665
13	Yilian Hydrologic Station	18.731	109.867	940	12.121	119.932
14	Back mountain of vacation village	18.733	109.861	1,130	21.561	109.599
15	Big Diaoluo	18.728	109.891	935	9.620	53.797

Tree sampling was carried out in 15 50 × 50 m sites with different latitudes, slopes, and aspects (direction that a slope faces, in angular degree) in the Diaoluo Mountain tropical forest in 2010 and 2015 separately. Each sampling site was divided into 25 contiguous 10 × 10 m plots. During each year and within each plot, individual trees with diameter at breast height (dbh) >2 centimeters (cm) were sampled. One sampled tree from each plot was selected randomly and measured by altimeter for its height (m). All other sampled trees from the same plot were compared with this measured tree visually, and their heights were estimated by field workers. For each sampled individual, its Latin name, dbh (cm), tree height (m), undercrown height (cm), crown diameter (cm) from west to east, and crown diameter (cm) from south to north were recorded.

To analyze the effect of individual variation at different taxonomic ranks, we classified each individual tree into species, genus, family, order, and superorder following the Angiosperm Phylogeny Group (APG III and APG IV) classification system (The Angiosperm Phylogeny Group, 2009; 2016). We retrieved these taxonomic names from the plant database Tropicos (www.tropicos.org) and added them into our raw data set. In total, our data contained 649 species, 255 genera, 91 families, 40 orders, and 11 superorders of tree taxa.

We calculated the aboveground biomass (AGB, in g) of each individual from its dbh (cm), height (converted to cm), and wood density (g/cm^3^) as

(5) $$\text{AGB}=0.4\pi {\left(\frac{\text{dbh}}{2}\right)}^{2}\times \left(\text{height + 300}\right)\times \left(\text{wood density}\right),$$

where 0.4 was the experimental form factor for fixed broadleaf forests in Hainan (Lin, 1964, 1974). The wood density of each species was obtained from the literature (Bao & Jiang, 1998; Cheng, Yang, & Liu, 1992; Drescher et al., 2016; IWICAF (Institute of Wood Industry, Chinese Academy of Forestry), 1982; Jiang, Cheng, & Yin, 2010; Zhu, Shi, Fang, Liu, & Ji, 2015) and the TRY trait database (https://www.try-db.org/TryWeb/Home.php, Kattge et al., 2011). For species whose wood density was not available (54 species), its density was approximated using that of a species from the same genus or family. For example, the wood density of *Hopea Chinese* was not available in the literature and was approximated using the wood density of *Vatica mangachapoi*, a species from the same family (Dipterocarpaceae) as *Hopea Chinese*. Three of the 54 species (four individuals) were the only member in their corresponding genus or family and were excluded from the analysis using AGB.

The tree sample data (Table S1, available on Dryad), with 14,904 individuals in 2010 and 14,658 individuals in 2015, were used to derive two data sets for analysis. The first data set included all individuals with positive dbh, positive height, positive wood density, and consequently positive AGB estimates. The number of deleted individuals in the first data set was 61 (61/14,904 ≈ 0.41%) in 2010 and 377 (377/14,658 ≈ 2.57%) in 2015. The second data set included all individuals with positive dbh measurements (all records with NA dbh were deleted). The number of deleted individuals in the second data set was 14 (14/14,904 ≈ 0.09%) in 2010 and 320 (320/14,658 ≈ 2.18%) in 2015. The discrepancy in the number of individuals between the two data sets for analysis was because some individuals (47 in 2010 and 57 in 2015) in the second data set (with positive dbh) did not have height or wood density records, and were therefore absent from the first data set. In a given year, the minimum number of individuals (regardless of taxon) in a plot was three, and the minimum number of individuals (regardless of taxon) in a site was 548. We used the first and second data sets to test the scaling relationships with AGB and dbh as individual size measure, respectively.

### 2.2 Community‐level analysis using taxon‐mixed data

We tested the scaling relationships (Equations (1), (2), (3), (4)) for taxon‐mixed data in each sampling year. In a given year, we defined the plot‐level assemblage abundance by tallying the number of individuals (regardless of taxon) within each plot from each site. We then calculated the spatial mean and the spatial variance of the assemblage abundance across all plots within each site. On the other hand, we calculated the mean and variance of individual body size (using AGB and dbh separately) across all individuals (regardless of taxon) within a site. Each of the 15 sites was associated with one quadruple of mean individual size, variance of individual size, spatial mean abundance, and spatial variance of abundance.

To test the power‐law patterns in Equations (1), (2), (3), (4) at the site scale, we fitted each bivariate relationship at doubly logarithmic scale. For example, when testing Taylor's law for assemblage abundance, we fitted log(variance of abundance) as a function of log(mean abundance) across the 15 sites using least‐squares linear regression. We fitted least‐squares quadratic regression to the same data to check if the relationship between log(mean abundance) and log(variance of abundance) was nonlinear (Taylor, Woiwod, & Perry, 1978).

(6) $$\begin{array}{cc} \text{log}\left(\text{variance of abundance}\right)\; & =\text{log}\left(c\right)+d\text{log}\left(\text{mean abundance}\right)\\ & +e{\left[\text{log}\left(\text{mean abundance}\right)\right]}^{2} \end{array}$$

If the slope of linear regression was significantly different from zero (95% confidence interval (CI) did not contain zero) and the quadratic coefficient of quadratic regression (*e* in Equation (6)) was not significantly different from zero (95% CI contained zero), then Taylor's law for assemblage abundance was not rejected (Table 2).

**Table 2:** Regression statistics of the four scaling relationships (Equations (1), (2), (3), (4)) fitted to taxon‐mixed data across 15 sampling sites, for each combination of sampling year (2010 and 2015) and individual size measure (aboveground biomass [AGB] [g] and diameter at breast height [dbh] [cm]) separately

Individual size measure	Scaling relationship	Year = 2010				Year = 2015			
		Slope of linear regression (95% CI)	Adj. *R* ^2^ of linear regression	Quadratic coefficient of quadratic regression (95% CI)	Adj. *R* ^2^ of quadratic regression	Slope of linear regression (95% CI)	Adj. *R* ^2^ of linear regression	Quadratic coefficient of quadratic regression (95% CI)	Adj. *R* ^2^ of quadratic regression
AGB	Taylor's law for individual size	2.6965 (2.2404, 3.1526)	0.9205	0.0123 (−1.4451, 1.4697)	0.9139	2.7069 (2.2107, 3.2030)	0.9078	−0.3067 (−1.9687, 1.3553)	0.9015
	Taylor's law for abundance	1.5718 (0.8245, 2.3192)	0.5839	−2.5490 (−7.0742, 1.9761)	0.5995	1.5988 (0.8898, 2.3078)	0.6189	−1.622 (−6.7011, 3.4576)	0.6031
	Abundance–size relationship	−0.3947 (−0.5842, −0.2052)	0.5788	0.1274 (−0.4729, 0.7277)	0.5517	−0.4317 (−0.6119, −0.2515)	0.6481	0.2216 (−0.3699, 0.8131)	0.6388
	Abundance–size variance relationship	−0.3027 (−0.4219, −0.1834)	0.675	−0.0518 (−0.1715, 0.0679)	0.6722	−0.2931 (−0.4281, −0.1581)	0.5999	−0.0604 (−0.2182, 0.0974)	0.5903
dbh	Taylor's law for individual size	4.1662 (3.1675, 5.1649)	0.8514	6.1810 (−7.0585, 19.4207)	0.8518	4.2539 (3.2761, 5.2317)	0.8619	5.1270 (−10.4291, 20.6830)	0.8565
	Taylor's law for abundance	1.6168 (0.8503, 2.3833)	0.5854	−2.803 (−7.4867, 1.8803)	0.6066	1.6463 (0.9262, 2.3663)	0.6256	−1.9920 (−7.1772, 3.1924)	0.6168
	Abundance–size relationship	−1.6799 (−2.3073, −1.0526)	0.6987	0.9411 (−7.7066, 9.5888)	0.6751	−1.7336 (−2.3241, −1.1431)	0.737	2.8110 (−6.6198, 12.2409)	0.7247
	Abundance–size variance relationship	−0.7732 (−1.0602, −0.4862)	0.7014	−0.3073 (−1.0019, 0.3874)	0.6998	−0.6996 (−1.0313, −0.3679)	0.5854	−0.2768 (−1.2687, 0.7151)	0.5642

In addition, we tested Taylor's law for individual size (Equation (2)) and abundance–size relationship (Equation (3)) at the plot scale. In Equation (2), mean and variance of individual size (using AGB and dbh separately) were calculated across all individuals (regardless of taxon) within a plot. In Equation (3), mean abundance was replaced by plot‐level abundance. Regression analysis performed at the site scale was repeated at the plot scale (Table S2). Taylor's law for abundance (Equation (1)) and abundance–size variance relationship (Equation (4)) were not testable at the plot scale, because they involved variance of abundance that can be calculated at the site scale only.

### 2.3 Population‐level analysis using taxon‐specific data

We repeated the above analyses for taxon‐specific individuals and populations of each species, genus, family, order, and superorder separately. We only tested each taxon with at least five mean–variance pairs of abundance or individual size, at the site and plot scale separately. The number of taxa tested for the four scaling relationships (Equations (1), (2), (3), (4)) and the proportion of nonsignificant linear regressions, at each combination of taxonomic rank (species, genus, family, order, and superorder), individual size measure (AGB and dbh), spatial scale (site and plot) were listed in Table 3 and Table S3. We calculated the Clopper–Pearson binomial 95% CI (Clopper & Pearson, 1934) of these proportions to check whether the observed significant linear regression can occur as random event. If the 95% CI contained 0.05, then it meant that those significant regressions occurred around 5% of the total regressions and maybe caused by chance alone. We calculated the average of adjusted coefficient of determination (adj. *R* ^2^) per linear regression across taxa at each taxonomic rank. Regression statistics and plots can be found in Tables [Link], [Link], [Link], [Link] and Figs S4–S58.

**Table 3:** Proportion of significant linear regressions fitted to taxon‐specific data for each of the four scaling relationships (Equations (1), (2), (3), (4)) at the site scale, under each combination of year, individual size measure, and taxonomic rank separately. Numbers in each parenthesis showed the 95% binomial confidence interval of the percentage of taxa with significant linear regression slopes. Second line in each cell gave the number of positive (+) and negative (−) linear relationships, as shown by the linear regressions

Scaling relationship	Year	Size measure	Species	Genus	Family	Order	Superorder
Taylor's law for individual size	2010	dbh	67/101 (0.56, 0.75)+99, −2	61/81 (0.64, 0.84) +79, −2	39/47 (0.69, 0.92) +46, −1	19/22 (0.65, 0.97) +22, −0	7/7 (0.59, 1) +7, −0
		AGB	89/101 (0.80, 0.94) +101, −0	71/81 (0.78, 0.94) +81, −0	45/47 (0.85, 0.99) +47, −0	22/22 (0.85, 1) +22, −0	7/7 (0.59, 1) +7, −0
	2015	dbh	57/98 (0.48, 0.68) +94, −4	57/79 (0.61, 0.82) +76, −3	41/47 (0.74, 0.95) +46, −1	20/22 (0.71, 0.99) +22, −0	7/7 (0.59, 1) +7, −0
		AGB	84/99 (0.76, 0.91) +99, −0	71/80 (0.80, 0.95) +79, −1	46/47 (0.89, 1) +46, −1	22/22 (0.85, 1) +22, −0	7/7 (0.59, 1) +7, −0
Taylor's law for abundance	2010	dbh	40/51 (0.65, 0.89) +49, −2	50/58 (0.75, 0.94) +57, −1	37/42 (0.74, 0.96) +41, −1	19/19 (0.82, 1) +19, −0	7/7 (0.59, 1) +7, −0
		AGB	40/51 (0.65, 0.89) +49, −2	50/58 (0.75, 0.94) +57, −1	37/42 (0.74, 0.96) +41, −1	19/19 (0.82, 1) +19, −0	7/7 (0.59, 1) +7, −0
	2015	dbh	35/45 (0.63, 0.89) +44, −1	45/53 (0.72, 0.93) +53, −0	37/40 (0.80, 0.98) +40, −0	19/19 (0.82, 1) +19, −0	7/7 (0.59, 1) +7, −0
		AGB	35/45 (0.63, 0.89) +44, −1	45/53 (0.72, 0.93) +53, −0	37/40 (0.80, 0.98) +40, −0	19/19 (0.82, 1) +19, −0	7/7 (0.59, 1) +7, −0
Abundance–size relationship	2010	dbh	3/51 (0.01, 0.16) +20, −31	2/58 (0.004, 0.12) +26, −32	6/42 (0.05, 0.29) +19, −23	3/19 (0.03, 0.40) +7, −12	4/7 (0.18, 0.90) +0, −7
		AGB	3/51 (0.01, 0.16) +21, −30	4/58 (0.02, 0.17) 25, −33	7/42 (0.07, 0.31) +17, −25	4/19 (0.06, 0.46) +8, −11	3/7 (0.10, 0.82) +1, −6
	2015	dbh	5/45 (0.04, 0.24) +15, −30	2/53 (0.005, 0.13) +22, −31	4/40 (0.03, 0.24) +16, −24	2/19 (0.01, 0.33) +4, −15	4/7 (0.18, 0.90) +0, −7
		AGB	6/45 (0.05, 0.27) +16, −29	5/53 (0.03, 0.21) +22, −31	6/40 (0.06, 0.30) +17, −23	4/19 (0.06, 0.46) +7, −12	4/7 (0.18, 0.90) +1, −6
Abundance–size variance relationship	2010	dbh	5/51 (0.03, 0.21) +22, −29	8/58 (0.06, 0.25) +23, −35	9/42 (0.10, 0.37) +18, −24	4/19 (0.06, 0.46) +5, −14	4/7 (0.18, 0.90) +1, −6
		AGB	4/51 (0.02, 0.19) +26, −25	8/58 (0.06, 0.25) +27, −31	8/42 (0.09, 0.34) +22, −20	3/19 (0.03, 0.40) +7, −12	4/7 (0.18, 0.90) +1, −6
	2015	dbh	4/45 (0.02, 0.21) +20, −25	6/53 (0.04, 0.23) +21, −32	7/40 (0.07, 0.33) +17, −23	3/19 (0.03, 0.40) +6, −13	4/7 (0.18, 0.90) +1, −6
		AGB	5/45 (0.04, 0.24) +24, −21	6/53 (0.04, 0.23) +24, −29	5/40 (0.04, 0.27) +20, −20	3/19 (0.03, 0.40) +6, −13	4/7 (0.18, 0.90) +1, −6

In addition to the analysis for each taxon individually, we tested the four scaling relationships (Equations (1), (2), (3), (4)) using lumped taxon‐specific means and variances from all taxa, at each rank separately. For example, at the species rank, when testing Taylor's law for individual size, we lumped the means and variances of individual body size (in AGB or dbh) of each species and fitted a linear regression and a quadratic regression to log(variance of individual size) as a function of log(mean individual size). We also fitted a Loess function (Cleveland & Devlin, 1988) to the lumped data to detect any trend. Regression statistics and plots for lumped data can be found in Tables [Link], [Link], [Link], [Link] and Figs S59–S70.

### 2.4 Effect of species richness on abundance–size variance relationship

To examine whether and how species richness changes the effect of individual size variation on spatial variation of abundance of community and population, we added species richness (number of distinct species) within a site to the abundance–size variance relationship (Equation (4)), using taxon‐mixed and taxon‐specific (lumped) data (at each taxonomic rank), respectively. On doubly logarithmic scale, we modified Equation (4) as

(7) $$\begin{array}{cc} & \text{log}\left(\text{variance of abundance}\right)\\ & \;=\;\text{log}\left(\gamma \right)+\eta \text{log}\left(\text{variance of individual size}\right)\\ & \;+\lambda \left(\text{species richness}\right)\\ & \;+\mu \left[\text{log}\left(\text{variance of individual size}\right)\text{:species richness}\right] \end{array}$$

In Equation (7), η was the power exponent of the abundance–size variance relationship (Equation (4)), λ and μ quantified, respectively, the effect of species richness on the intercept and slope of abundance–size variance relationship. For example, if μ was significantly larger than zero, then it meant that greater species richness increased the slope of abundance–size variance relationship. We fitted Equation (7) using AGB and dbh as individual size measure separately.

In this work, log = log_10_ unless specified otherwise. Significance level of a hypothesis test was set at 0.05. Least‐squares regressions and confidence intervals were done in R 3.4.0 (R Core Team 2017).

### 2.5 Relation between scaling parameters under different size measures

Taylor's law for individual size, abundance–size relationship, and abundance–size variance relationship involved variables at the individual level. In our empirical analysis, each of the above relationships was tested with AGB and dbh as size measure separately. Using the delta method (Cramér, 1946; Oehlert, 1992), the variance of product formula (Goodman 1960), the biomass equation (Equation (5)), and the allometry between tree height and dbh, for each of the three scaling relationships, we derived an analytic formula linking its power exponent estimated under different individual size measures. We tested our theory using community‐level data from the Diaoluo Mountain.

**Figure 2:**
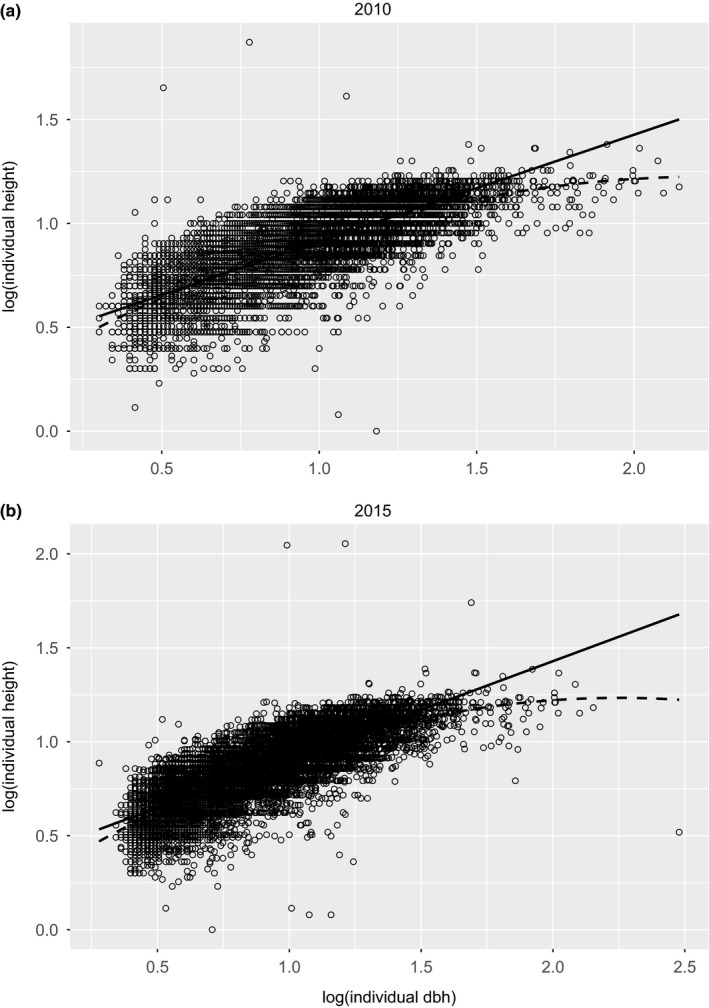
Log(individual height) plotted against log(individual dbh) across all trees in (a) 2010 and (b) 2015 separately. Solid and dashed lines were fitted linear and quadratic regression lines, respectively. The linear regression equations and parameter confidence intervals (in parenthesis) were log(individual height) = 0.3980 (0.3929, 0.4032) + 0.5146 (0.5085, 0.5207) × log(individual dbh) in 2010 and log(individual height) = 0.3890 (0.3836, 0.3944) + 0.5199 (0.5137, 0.5261) × log(individual dbh) in 2015. The quadratic regression equations and parameter confidence intervals (in parenthesis) were log(individual height) = 0.2590 (0.2457, 0.2723) + 0.8613 (0.8300, 0.8926) × log(individual dbh) − 0.1919 (−0.2089, −0.1749) × [log(individual dbh)]^2^ in 2010 and log(individual height) = 0.2392 (0.2251, 0.2533) + 0.8830 (0.8507, 0.9152) × log(individual dbh) − 0.1960 (−0.2131, −0.1789) × [log(individual dbh)]^2^ in 2015

We analyzed the allometry between tree height and dbh at individual level. We fitted a least‐square linear regression to log(individual height) as a function of log(individual dbh) across all sampled individuals in each year and used its slope as the exponent estimate of the allometric relationship. We also fitted a quadratic regression to examine the curvature between log(individual height) and log(individual dbh) (Figure 2).

## 3 RESULTS

### 3.1 Taxon‐mixed scaling

**Figure 3:**
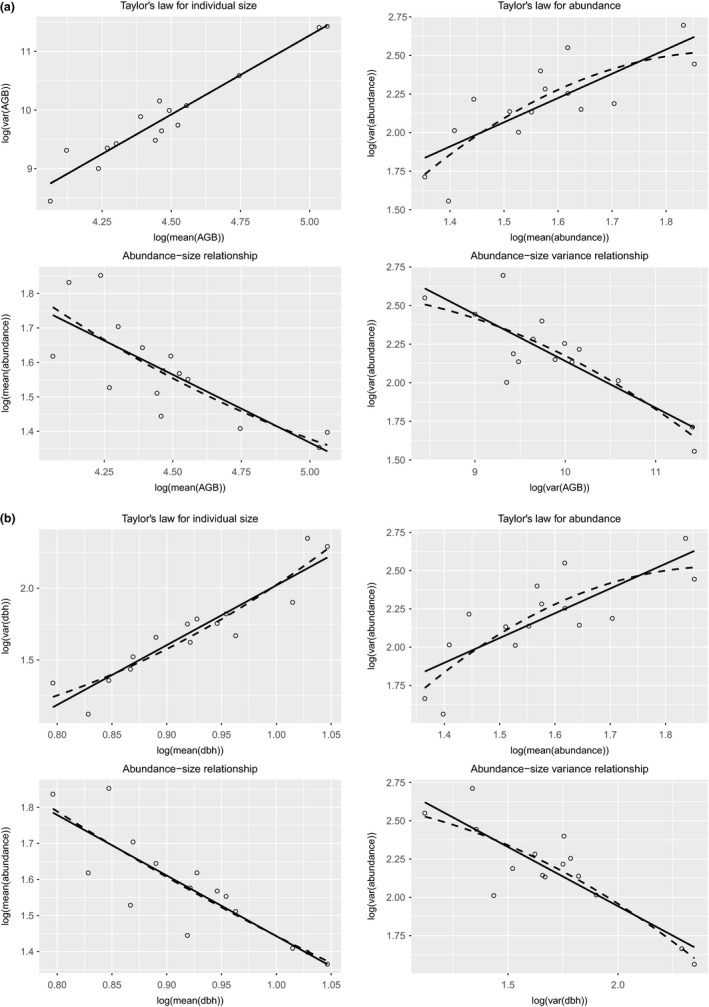
Four scaling relationships for taxon‐mixed data in 2010 using (a) AGB and (b) dbh as size measure separately, with one circle per site. Solid line and dashed line in each panel were the least‐squares linear and quadratic regression lines, respectively. Regression statistics were reported in Table 2

At the site level, power‐law pattern of the four scaling relationships (Equations (1), (2), (3), (4)) was confirmed, under each combination of year and size measure. Specifically, linear regressions fitted to Taylor's law for abundance and individual size showed significantly positive slopes; linear regressions fitted to abundance–size relationship and abundance–size variance relationship showed significantly negative slopes. Quadratic coefficient of each fitted quadratic regression was not significantly different from zero in any relationship (Figure 3 and Fig. S2, Table 2).

Moreover, empirical estimates of the power exponents of Taylor's law for abundance, Taylor's law for individual size, and abundance–size relationship predicted reasonably the power exponent of abundance–size variance relationship (Equation (4)). Specifically, predicted exponent of the abundance–size variance relationship ([−0.3947] × 1.5718/2.6965 ≈ −0.2301 for AGB in 2010; [−0.4317] × 1.5988/2.7069 ≈ −0.2550 for AGB in 2015; [−1.6799] × 1.6168/4.1662 ≈ −0.6519 for dbh in 2010; [−1.7336] × 1.6463/4.2539 ≈ −0.6709 for dbh in 2015) fell within the corresponding 95% confidence interval (CI) estimated from data ([−0.4219, −0.1834] for AGB in 2010; [−0.4281, −0.1581] for AGB in 2015; [−1.0602, −0.4862] for dbh in 2010; [−1.0313, −0.3679] for dbh in 2015) (Table 2).

At the plot scale, slope of the linear regression fitted to Taylor's law for individual size was significantly positive, under each combination of year and size measure. Quadratic coefficient of the quadratic regression was significantly positive for AGB in 2015 and significantly negative for dbh in 2010. For abundance–size relationship, under each combination of year and size measure, slope of the fitted linear regression was significantly negative, and quadratic coefficient of the quadratic regression was not different from zero (Figs S2 and S3, Table S2).

### 3.2 Taxon‐specific scaling

At the site scale, for majority of taxa tested individually, linear relationship between log(mean abundance) (or log(mean individual size)) and log(variance of abundance) (or log(variance of individual size)) was significant and positive, regardless of sampling year, size measure, and spatial scale (Table 3). This indicated that Taylor's law for abundance and Taylor's law for individual size were reasonable models for the corresponding mean–variance relationship. For taxa with significant linear regressions, across years and taxonomic ranks, the slope estimates ranged from 1.07 to 5.38 in Taylor's law for abundance, and from 1.15 to 9.62 and from 1.27 to 10.42 in Taylor's law for individual size with AGB and dbh as respective size measure. Average adj. *R* ^2^ ranged from 0.71 to 0.82 in Taylor's law for abundance, and from 0.78 to 0.92 and from 0.50 to 0.75 in Taylor's law for individual size with AGB and dbh as respective size measure (Tables S4 and S6). On the other hand, linear regression fitted to taxon‐specific abundance–size relationship, and abundance–size variance relationship was significant with small probabilities, mostly not or only marginally different from 0.05, as shown by the 95% binomial confidence interval (Table 3). For both relationships, the sign of fitted regression was neither uniformly positive nor uniformly negative. Average adj. *R* ^2^ ranged from 0.02 to 0.34 and from 0.01 to 0.37 in abundance–size relationship with AGB and dbh as respective size measure and from 0.04 to 0.34 and from 0.04 to 0.35 in abundance–size variance relationship with AGB and dbh as respective size measure, across years and taxonomic ranks (Tables S4 and S6). Analysis of Taylor's law for individual size and abundance–size relationship at the plot scale reached the same conclusion (Tables S3, S5, and S7).

Using lumped taxon‐specific means and variances, under each combination of year and taxonomic rank, the slope of linear regression was significantly positive in Taylor's law for abundance and Taylor's law for individual size, but not significantly different from zero in abundance–size relationship and abundance–size variance relationship at the site scale, agreeing with the prediction from Equation (4) (β*b*/*d *=* *0 when β = 0). Quadratic regression (Equation (6)) and Loess function showed significant concavity on doubly logarithmic scale, rejecting the power‐law pattern in each scaling relationship (Equations (1), (2), (3), (4)) (Tables S8 and S10). Taylor's law for individual size and abundance–size relationship at the plot scale showed the same conclusion (Tables S9 and S11).

### 3.3 Species richness has weak effect on abundance variation at population and community levels

At the community level, species richness did not show significant effect on the slope or the intercept of abundance–size variance relationship, regardless of year and size measure. Adding species richness to the model (Equation (7)) made the effect of variance of individual size insignificant in the abundance–size variance relationship. Adj. *R* ^2^ was not substantially different with or without species richness in the model.

At the population level, regardless of year, size measure and taxonomic rank, inclusion of species richness in the abundance–size variance relationship did not change the observation that individual size variation had no significant effect on spatial variation of taxon‐specific population abundance. In addition, species richness did not significantly change the intercept or slope of abundance–size variance relationship, nor did it change substantially the adj *R* ^2^.

### 3.4 Allometric theory links exponents of scaling relationship under different individual size measures

We showed that, for each of the three scaling relationships (Taylor's law for individual size, abundance–size relationship, and abundance–size variance relationship), the power exponent estimated under different individual size measures (AGB and dbh) was related analytically. Specifically, denoting the power exponents of Taylor's law for individual size, abundance–size relationship, and abundance–size variance relationship, respectively, as *d*_AGB_, β_AGB_, and η_AGB_ when AGB was the size measure, and as *d*_dbh_, β_dbh_, and η_dbh_ when dbh was the size measure, we obtained

(8) $$d_{\mathrm{AGB}}\approx \frac{2g+d_{\mathrm{dbh}}+2}{g+2},$$

(9) $$\beta_{\mathrm{AGB}}\approx \frac{\beta_{\mathrm{dbh}}}{g+2},$$

and

(10) $$\eta_{\mathrm{AGB}}\approx \frac{d_{\mathrm{dbh}}\times \eta_{\mathrm{dbh}}}{2g+d_{\mathrm{dbh}}+2}.$$

Here *g* is the power‐law exponent of height‐dbh allometry estimated from linear regression (see Figure 2 legend). Using taxon‐mixed data at the site scale in Diaoluo Mountain, we found that *d*_AGB_ predicted from Equation (8) was not significantly different from the corresponding value estimated from data; but β_AGB_ and η_AGB_ predicted from Equations (9) and (10), respectively, were significantly different from the corresponding values estimated from data. Analytic derivations of Equations (8), (9), (10) and their empirical testing were detailed in the Appendix.

## 4 DISCUSSION

To summarize our findings here, we used Taylor's law and abundance–size relationship to derive a new scaling pattern (called abundance–size variance relationship) relating individual size variation to spatial variation of abundance. The power‐law scaling framework was confirmed for taxon‐mixed plant communities, but not for taxon‐specific plant populations, under different spatial scales (site and plot) and individual size measures (AGB and dbh) separately. Based on our theoretical framework, the community‐level spatial variation of assemblage abundance was negatively correlated with the individual size variation in a power‐law form, of which the power exponent can be predicted from Taylor's law and abundance–size relationship. The lack of power‐law relationship between individual size variation and spatial variation of population abundance can be attributed to the weak abundance–size relationship for taxon‐specific populations. Species richness did not change the intercept or the slope of abundance–size variance relationship, regardless of individual size measures and taxonomic ranks. Negative abundance–size variance relationship at the community level suggested that interindividual variation of body size dampens the spatial variation of community assemblage abundance.

A central question arisen from our results is: Why did the scaling relationships (Equations (1), (2), (3), (4)) show different patterns at population and community levels?

**Figure 4:**
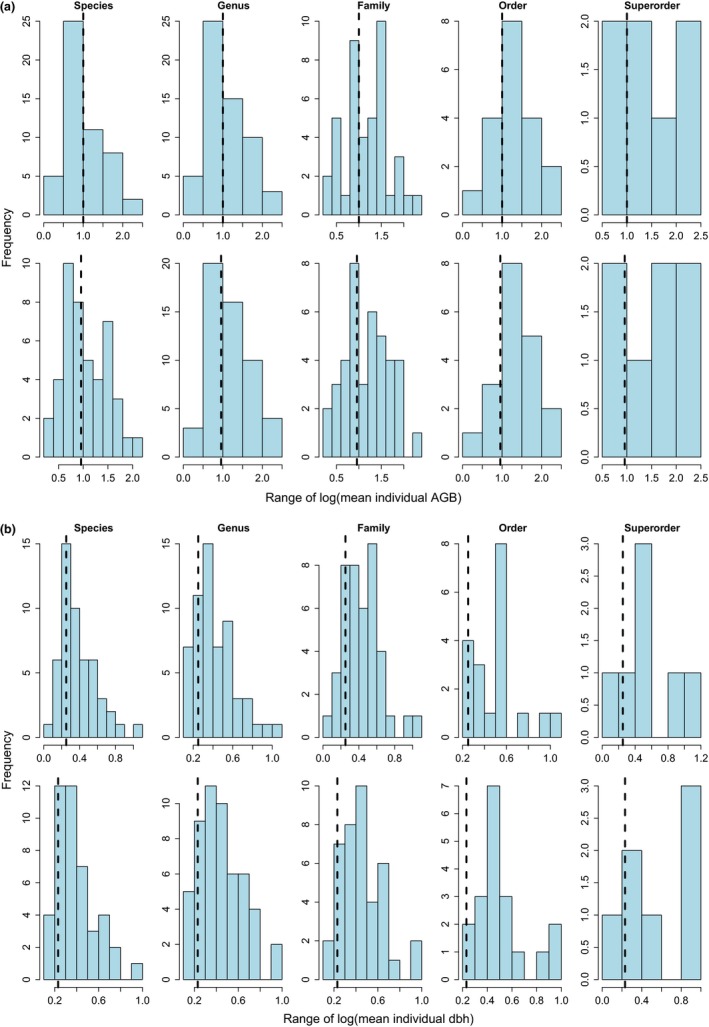
Comparison of ranges of log(mean individual size) at the site scale between taxon‐specific population data and taxon‐mixed community data, using (a) AGB and (b) dbh as size measure separately. Histogram in each panel showed the frequency distribution of the range of log(mean individual size) per taxon at each rank in 2010 (top row) and 2015 (bottom row) separately. Dashed vertical line was the range of log(mean individual size) for the community data. Range was calculated as the maximum log(mean individual size) within a site minus the minimum log(mean individual size) within a site (for each taxon or regardless of taxon)

A statistical reason may contribute to the lack of negative power‐law relationship between taxon‐specific mean (or variance of) individual size and spatial mean (or spatial variance of) abundance. That is, taxon‐specific individuals may show limited size variation that can hide the true relationship from detection. However, empirical evidence from the current work was against such claim. First, comparison of size variation among taxon‐specific individuals (at each taxonomic rank) and among taxon‐mixed individuals did not show substantial difference, regardless of year and size measure (Figure 4), probably due to the averaging effect among taxa. In particular, community‐level range of log(mean individual size) fell within the corresponding 95% confidence interval of population‐level range of log(mean individual size) (results not shown). Second, lumping taxon‐specific means and variances across taxa enlarged the range of individual size and its variation, but failed to produce a negative power‐law relationship as expected (Figs S61, S62, S64, S67, S68, and S70). Biological mechanisms must be at work to explain the observed discrepancy between population and community.

At the population level, abundance–size relationship (and abundance–size variance relationship) for single taxon yielded different signs. This may be attributed to the taxonomic variation in resource requirement and acquisition, where positive relationship showed taxon's ability of adapting to the local habitat and exploiting its ecological niche, and negative relationship indicated that taxon's spatial spread was refrained by local resources. It may also reflect the taxon's demographic difference caused by high species turnover in diverse communities (Allan et al., 2011), where positive relationship suggested that taxon was at its early development of growth, and negative relationship suggested that taxon entering mature or old status was regulated by self‐thinning (Mohler, Marks, & Sprugel, 1978).

The negative effect of individual size variation on spatial variation of assemblage abundance at the community level can be explained by the intertaxonomic competition through portfolio effect (Bolnick et al., 2011). Suppose the abundance of taxon *i* was *N*_*i*_ (*i *=* *1, 2, …, *S*), where *S* was the number of taxa within the community. Then, according to the formula for the variance of the sum of correlated random variables, the variance of assemblage abundance *N* (=$\sum_{i=1}^{S}N_{i}$ ) was

$$\text{var}\left(N\right)=\text{var}\left(\sum_{i=1}^{S}N_{i}\right)=\sum_{i\neq 1}^{S}\mathrm{var}\left(N_{i}\right)+\sum_{i\neq j}^{S}\text{cov}\left(N_{i},N_{j}\right).$$

Following our empirical result at the population level, *var*(*N*_*i*_) was independent of individual size variation. As more taxa were included, individual size variation increased due to intertaxonomic variation, and $\sum_{i=1}^{S}\text{var}\left(N_{i}\right)$ increased. On the other hand, negative density covariance ($\text{cov}\left(N_{i},N_{j}\right)$ ) between competing taxa reduced the overall variance of assemblage abundance. The negative power‐law pattern observed in the abundance–size variance relationship at the community level reflected that negative density dependence induced by intertaxonomic competition was stronger than the positive additive effect of taxonomic variation in individual size.

The analytic relationship of scaling parameters estimated using different size measures (AGB and dbh) can be derived for other biomass equations (Chave et al., 2005). For example, the general model I in Chave et al. (2005) stated that AGB was proportional to the product of wood density, dbh squared, and height. Their model differed from our biomass equation (Equation (5)) only in that the former did not have the adjusting constant for height (300 in Equation (5)). This difference did not alter the analytic formulas (Equations (8), (9), (10)) or their predictions. This suggested that the scaling framework developed here is robust to the particular form of biomass equations and is an intrinsic property of the plan community. On the other hand, the general model II in Chave et al. (2005) was based on the polynomial allometric relationship between log(height) and log(dbh) (Niklas, 1995). Interestingly, we observed similar pattern in the height‐dbh allometry using the Diaoluo Mountain data (Figure 2). It is worth investigating the analytic relation of scaling parameters when the height‐dbh allometry deviates from the power law. We leave this possibility as a research topic in the future.

The idea of integrating established scaling patterns to create a new pattern has been proposed (Marquet et al., 2005) and tested (Cohen, Xu, & Schuster, 2012; Lagrue, Poulin, & Cohen, 2015) previously. In Cohen et al. (2012), the authors used Taylor's law and abundance–size relationship to derive a new scaling relationship between the individual mean body mass and population abundance variance, called variance‐mass allometry. The analytic difference between variance‐mass allometry and abundance–size variance relationship examined here was elaborated using a conceptual probability distribution model in Xu (2016). Compared to the previous meta‐analysis (Xu, 2016), the current work provided an in‐depth analysis of the scaling relationships using a comprehensive plant data set. The plot‐site data structure allowed the first empirical testing of a spatial abundance–size variance relationship up to date. In addition, we were able to, for the first time, compare the effects of individual size variation on spatial variation of abundance between population and community levels. Findings from this work improved our understanding of the mechanisms of spatial variation of tropical plant population and community, which can provide insights into the management and conservation of the forest biodiversity and productivity.

## 5 DATA ACCESSIBILITY

Diaoluo Mountain tree sample data are available on Dryad (https://doi.org/10.5061/dryad.87n81).

## AUTHOR CONTRIBUTIONS

Wang and Xu designed the research and wrote the first draft of the manuscript. Xu analyzed the data and revised the manuscript.

## CONFLICT OF INTEREST

None declared.
