# Supplementary material for: Individual size variation reduces spatial variation in abundance of tree community assemblage, not of tree populations
Source: Ecol Evol. 2017 Nov 9;7(24):10815–28. doi: 10.1002/ece3.3594 (PMC5743614; doi:10.1002/ece3.3594)
Supplement: Supplementary file 1 [file ECE3-7-10815-s001.docx]

Individual size variation reduces spatial variation in abundance of tree community assemblage, not of tree populations

Hua-Feng Wang^1^, Meng Xu^2,*^

^1^Hainan Key Laboratory for Sustainable Utilization of Tropical Bioresources, Institute of Tropical Agriculture and Forestry, Hainan University, Haikou, 570228, China

^2^Department of Mathematics, Pace University, 41 Park Row, New York, NY 10038, USA

^*^Corresponding author: Meng Xu, Department of Mathematics, Pace University, 41 Park Row, New York, NY 10038, USA. Email: [mxu@pace.edu](mailto:mxu@pace.edu)

Running Head: Scaling individual size and abundance variations

**Supplementary information**

*Supplementary tables*

Table S1. Regression statistics of Taylor's law for individual size and abundance-size relationship (eqns 2 and 3) fitted to taxon-mixed data across 375 plots (15 sites × 25 plots per site), for each combination of sampling year (2010 and 2015) and individual size measure (aboveground biomass (AGB) (g) and diameter at breast height (dbh) (cm)) separately

Table S2. Proportion of significant linear regressions fitted to taxon-specific data for Taylor's law for individual size and abundance-size relationship (eqns 2 and 3) at the plot scale, under each combination of year, individual size measure, and taxonomic rank separately. Second line in each cell showed the 95% binomial confidence interval of the percentage of taxa with significant linear regression slopes. Third line in each cell gave the number of positive (+) and negative (-) linear relationships, as shown by the linear regressions

Table S3. Regression statistics of four scaling relationships (eqns 1-4) fitted to taxon-specific data for each taxon at the site scale, with AGB as the individual size measure

Table S4. Regression statistics of Taylor's law for individual size and abundance-size relationship (eqns 2 and 3) fitted to taxon-specific data for each taxon at the plot scale, with AGB as the individual size measure

Table S5. Regression statistics of four scaling relationships (eqns 1-4) fitted to taxon-specific data for each taxon at the site scale, with dbh as the individual size measure

Table S6. Regression statistics of Taylor's law for individual size and abundance-size relationship (eqns 2 and 3) fitted to taxon-specific data for each taxon at the plot scale, with dbh as the individual size measure

Table S7. Regression statistics of four scaling relationships (eqns 1-4) fitted to taxon-specific data lumped from all taxa at each rank at the site scale, with AGB as the individual size measure

Table S8. Regression statistics of Taylor's law for individual size and abundance-size relationship (eqns 2 and 3) fitted to taxon-specific data lumped from all taxa at each rank at the plot scale, with AGB as the individual size measure

Table S9. Regression statistics of four scaling relationships (eqns 1-4) fitted to taxon-specific data lumped from all taxa at each rank at the site scale, with dbh as the individual size measure

Table S10. Regression statistics of Taylor's law for individual size and abundance-size relationship (eqns 2 and 3) fitted to taxon-specific data lumped from all taxa at each rank at the plot scale, with dbh as the individual size measure

*Supplementary figures and figure legends* **Figure S1**. Four scaling relationships for taxon-mixed data in 2015 using (a) AGB and (b) dbh as size measure separately, with one circle per site. Solid line and dashed line in each panel were the least-squares linear and quadratic regression lines respectively. Regression statistics were reported in Table 2.

 **Figure S2**. Taylor's law for individual size and abundance-size relationship for taxon-mixed data in 2010 (a and c) and 2015 (b and d) using AGB as size measure, with one circle per plot. Solid and dashed lines were defined in Fig. S1. **Figure S3**. Taylor's law for individual size and abundance-size relationship for taxon-mixed data in 2010 (a and c) and 2015 (b and d) using dbh as size measure, with one circle per plot. Solid and dashed lines were defined in Fig. S1.

 **Figure S4**. Log(variance of abundance) plotted against log(mean abundance) per site for each single species population in 2010 (a) and 2015 (b) separately. Solid line was the linear regression line.

 **Figure S5**. Log(variance of abundance) plotted against log(mean abundance) per site for each single genus population in 2010 (a) and 2015 (b) separately. Solid line was the linear regression line.

 **Figure S6**. Log(variance of abundance) plotted against log(mean abundance) per site for each single family population in 2010 (a) and 2015 (b) separately. Solid line was the linear regression line.

 **Figure S7**. Log(variance of abundance) plotted against log(mean abundance) per site for each single order population in 2010 (a) and 2015 (b) separately. Solid line was the linear regression line.

 **Figure S8**. Log(variance of abundance) plotted against log(mean abundance) per site for each single superorder population in 2010 (a) and 2015 (b) separately. Solid line was the linear regression line.

 **Figure S9**. Log(variance of individual AGB) plotted against log(mean individual AGB) per site for each single species in 2010 (a) and 2015 (b) separately. Solid line was the linear regression line.

 **Figure S10**. Log(variance of individual AGB) plotted against log(mean individual AGB) per site for each single genus in 2010 (a) and 2015 (b) separately. Solid line was the linear regression line.

 **Figure S11**. Log(variance of individual AGB) plotted against log(mean individual AGB) per site for each single family in 2010 (a) and 2015 (b) separately. Solid line was the linear regression line.

 **Figure S12**. Log(variance of individual AGB) plotted against log(mean individual AGB) per site for each single order in 2010 (a) and 2015 (b) separately. Solid line was the linear regression line.

 **Figure S13**. Log(variance of individual AGB) plotted against log(mean individual AGB) per site for each single superorder in 2010 (a) and 2015 (b) separately. Solid line was the linear regression line.

 **Figure S14**. Log(mean abundance) plotted against log(mean individual AGB) per site for each single species in 2010 (a) and 2015 (b) separately. Solid line was the linear regression line.

 **Figure S15**. Log(mean abundance) plotted against log(mean individual AGB) per site for each single genus in 2010 (a) and 2015 (b) separately. Solid line was the linear regression line.

 **Figure S16**. Log(mean abundance) plotted against log(mean individual AGB) per site for each single family in 2010 (a) and 2015 (b) separately. Solid line was the linear regression line.

 **Figure S17**. Log(mean abundance) plotted against log(mean individual AGB) per site for each single order in 2010 (a) and 2015 (b) separately. Solid line was the linear regression line.

 **Figure S18**. Log(mean abundance) plotted against log(mean individual AGB) per site for each single superorder in 2010 (a) and 2015 (b) separately. Solid line was the linear regression line.

 **Figure S19**. Log(variance of abundance) plotted against log(variance of individual AGB) per site for each single species in 2010 (a) and 2015 (b) separately. Solid line was the linear regression line.

 **Figure S20**. Log(variance of abundance) plotted against log(variance of individual AGB) per site for each single genus in 2010 (a) and 2015 (b) separately. Solid line was the linear regression line.

 **Figure S21**. Log(variance of abundance) plotted against log(variance of individual AGB) per site for each single family in 2010 (a) and 2015 (b) separately. Solid line was the linear regression line.

 **Figure S22**. Log(variance of abundance) plotted against log(variance of individual AGB) per site for each single order in 2010 (a) and 2015 (b) separately. Solid line was the linear regression line.

 **Figure S23**. Log(variance of abundance) plotted against log(variance of individual AGB) per site for each single superorder in 2010 (a) and 2015 (b) separately. Solid line was the linear regression line.

**Figure S24**. Log(variance of individual AGB) plotted against log(mean individual AGB) per plot for each single species in 2010 (a) and 2015 (b) separately. Solid line was the linear regression line.

 **Figure S25**. Log(variance of individual AGB) plotted against log(mean individual AGB) per plot for each single genus in 2010 (a) and 2015 (b) separately. Solid line was the linear regression line.

**Figure S26**. Log(variance of individual AGB) plotted against log(mean individual AGB) per plot for each single family in 2010 (a) and 2015 (b) separately. Solid line was the linear regression line.

 **Figure S27**. Log(variance of individual AGB) plotted against log(mean individual AGB) per plot for each single order in 2010 (a) and 2015 (b) separately. Solid line was the linear regression line.

 **Figure S28**. Log(variance of individual AGB) plotted against log(mean individual AGB) per plot for each single superorder in 2010 (a) and 2015 (b) separately. Solid line was the linear regression line.

 **Figure S29**. Log(abundance) plotted against log(mean individual AGB) per plot for each single species in 2010 (a) and 2015 (b) separately. Solid line was the linear regression line.

 **Figure S30**. Log(abundance) plotted against log(mean individual AGB) per plot for each single genus in 2010 (a) and 2015 (b) separately. Solid line was the linear regression line.

 **Figure S31**. Log(abundance) plotted against log(mean individual AGB) per plot for each single family in 2010 (a) and 2015 (b) separately. Solid line was the linear regression line.

 **Figure S32**. Log(abundance) plotted against log(mean individual AGB) per plot for each single order in 2010 (a) and 2015 (b) separately. Solid line was the linear regression line.

 **Figure S33**. Log(abundance) plotted against log(mean individual AGB) per plot for each single superorder in 2010 (a) and 2015 (b) separately. Solid line was the linear regression line.

 **Figure S34**. Log(variance of individual dbh) plotted against log(mean individual dbh) per site for each single species in 2010 (a) and 2015 (b) separately. Solid line was the linear regression line.

 **Figure S35**. Log(variance of individual dbh) plotted against log(mean individual dbh) per site for each single genus in 2010 (a) and 2015 (b) separately. Solid line was the linear regression line.

 **Figure S36**. Log(variance of individual dbh) plotted against log(mean individual dbh) per site for each single family in 2010 (a) and 2015 (b) separately. Solid line was the linear regression line.

 **Figure S37**. Log(variance of individual dbh) plotted against log(mean individual dbh) per site for each single order in 2010 (a) and 2015 (b) separately. Solid line was the linear regression line.

 **Figure S38**. Log(variance of individual dbh) plotted against log(mean individual dbh) per site for each single superorder in 2010 (a) and 2015 (b) separately. Solid line was the linear regression line.

 **Figure S39**. Log(mean abundance) plotted against log(mean individual dbh) per site for each single species in 2010 (a) and 2015 (b) separately. Solid line was the linear regression line.

 **Figure S40**. Log(mean abundance) plotted against log(mean individual dbh) per site for each single genus in 2010 (a) and 2015 (b) separately. Solid line was the linear regression line.

 **Figure S41**. Log(mean abundance) plotted against log(mean individual dbh) per site for each single family in 2010 (a) and 2015 (b) separately. Solid line was the linear regression line.

 **Figure S42**. Log(mean abundance) plotted against log(mean individual dbh) per site for each single order in 2010 (a) and 2015 (b) separately. Solid line was the linear regression line.

 **Figure S43**. Log(mean abundance) plotted against log(mean individual dbh) per site for each single superorder in 2010 (a) and 2015 (b) separately. Solid line was the linear regression line.

 **Figure S44**. Log(variance of abundance) plotted against log(variance of individual dbh) per site for each single species in 2010 (a) and 2015 (b) separately. Solid line was the linear regression line.

 **Figure S45**. Log(variance of abundance) plotted against log(variance of individual dbh) per site for each single genus in 2010 (a) and 2015 (b) separately. Solid line was the linear regression line.

 **Figure S46**. Log(variance of abundance) plotted against log(variance of individual dbh) per site for each single family in 2010 (a) and 2015 (b) separately. Solid line was the linear regression line.

 **Figure S47**. Log(variance of abundance) plotted against log(variance of individual dbh) per site for each single order in 2010 (a) and 2015 (b) separately. Solid line was the linear regression line.

 **Figure S48**. Log(variance of abundance) plotted against log(variance of individual dbh) per site for each single superorder in 2010 (a) and 2015 (b) separately. Solid line was the linear regression line.

 **Figure S49**. Log(variance of individual dbh) plotted against log(mean individual dbh) per plot for each single species in 2010 (a) and 2015 (b) separately. Solid line was the linear regression line.

 **Figure S50**. Log(variance of individual dbh) plotted against log(mean individual dbh) per plot for each single genus in 2010 (a) and 2015 (b) separately. Solid line was the linear regression line.

 **Figure S51**. Log(variance of individual dbh) plotted against log(mean individual dbh) per plot for each single family in 2010 (a) and 2015 (b) separately. Solid line was the linear regression line.

**Figure S52**. Log(variance of individual dbh) plotted against log(mean individual dbh) per plot for each single order in 2010 (a) and 2015 (b) separately. Solid line was the linear regression line.

**Figure S53**. Log(variance of individual dbh) plotted against log(mean individual dbh) per plot for each single superorder in 2010 (a) and 2015 (b) separately. Solid line was the linear regression line.

**Figure S54**. Log(abundance) plotted against log(mean individual dbh) per plot for each single species in 2010 (a) and 2015 (b) separately. Solid line was the linear regression line.

**Figure S55**. Log(abundance) plotted against log(mean individual dbh) per plot for each single genus in 2010 (a) and 2015 (b) separately. Solid line was the linear regression line.

**Figure S56**. Log(abundance) plotted against log(mean individual dbh) per plot for each single family in 2010 (a) and 2015 (b) separately. Solid line was the linear regression line.

**Figure S57**. Log(abundance) plotted against log(mean individual dbh) per plot for each single order in 2010 (a) and 2015 (b) separately. Solid line was the linear regression line.

**Figure S58**. Log(abundance) plotted against log(mean individual dbh) per plot for each single superorder in 2010 (a) and 2015 (b) separately. Solid line was the linear regression line.

**Figure S59**. Log(variance of individual AGB) plotted against log(mean individual AGB) per site using lumped taxon-specific data at each rank in 2010 (a) and 2015 (b) separately. Solid line was the linear regression line. Dashed line was the quadratic regression line. Dotted line was the fitted Loess function.

**Figure S60**. Log(variance of abundance) plotted against log(mean abundance) per site using lumped taxon-specific data at each rank in 2010 (a) and 2015 (b) separately. Each individual had positive AGB. Line styles were defined in Fig. S59.

**Figure S61**. Log(mean abundance) plotted against log(mean individual AGB) per site using lumped taxon-specific data at each rank in 2010 (a) and 2015 (b) separately. Line styles were defined in Fig. S59.

**Figure S62**. Log(variance of abundance) plotted against log(variance of individual AGB) per site using lumped taxon-specific data at each rank in 2010 (a) and 2015 (b) separately. Line styles were defined in Fig. S59.

**Figure S63**. Log(variance of individual AGB) plotted against log(mean individual AGB) per plot using lumped taxon-specific data at each rank in 2010 (a) and 2015 (b) separately. Line styles were defined in Fig. S59.

**Figure S64**. Log(abundance) plotted against log(mean individual AGB) per plot using lumped taxon-specific data at each rank in 2010 (a) and 2015 (b) separately. Line styles were defined in Fig. S59.

**Figure S65**. Log(variance of individual dbh) plotted against log(mean individual dbh) per site using lumped taxon-specific data at each rank in 2010 (a) and 2015 (b) separately. Line styles were defined in Fig. S59.

**Figure S66**. Log(variance of abundance) plotted against log(mean abundance) per site using lumped taxon-specific data at each rank in 2010 (a) and 2015 (b) separately. Each individual had positive dbh. Line styles were defined in Fig. S59.

**Figure S67**. Log(mean abundance) plotted against log(mean individual dbh) per site using lumped taxon-specific data at each rank in 2010 (a) and 2015 (b) separately. Line styles were defined in Fig. S59.

**Figure S68**. Log(variance of abundance) plotted against log(variance of individual dbh) per site using lumped taxon-specific data at each rank in 2010 (a) and 2015 (b) separately. Line styles were defined in Fig. S59.

**Figure S69**. Log(variance of individual dbh) plotted against log(mean individual dbh) per plot using lumped taxon-specific data at each rank in 2010 (a) and 2015 (b) separately. Line styles were defined in Fig. S59.

**Figure S70**. Log(abundance) plotted against log(mean individual dbh) per plot using lumped taxon-specific data at each rank in 2010 (a) and 2015 (b) separately. Line styles were defined in Fig. S59.
