# Supplementary material for: Individual size variation reduces spatial variation in abundance of tree community assemblage, not of tree populations
Source: Ecol Evol. 2017 Nov 9;7(24):10815–28. doi: 10.1002/ece3.3594 (PMC5743614; doi:10.1002/ece3.3594)
Supplement: Supplementary file 13 [file ECE3-7-10815-s013.docx]

Individual size variation reduces spatial variation in abundance of tree community assemblage, not of tree populations

Hua-Feng Wang^1^, Meng Xu^2,*^

^1^Hainan Key Laboratory for Sustainable Utilization of Tropical Bioresources, Institute of Tropical Agriculture and Forestry, Hainan University, Haikou, 570228, China

^2^Department of Mathematics, Pace University, 41 Park Row, New York, NY 10038, USA

^*^Corresponding author: Meng Xu, Department of Mathematics, Pace University, 41 Park Row, New York, NY 10038, USA. Email: [mxu@pace.edu](mailto:mxu@pace.edu)

Running Head: Scaling individual size and abundance variations

**Appendix**

In this appendix we derived the analytic relationship of the power exponent of each scaling relationship (Taylor's law for individual size, abundance-size relationship, abundance-size variance relationship) under different size measures (AGB and dbh).

*Relation of power exponent of Taylor's law for individual size*

Suppose Taylor's law for individual size (eqn 2) holds with dbh as the individual size measure (*V* and *E* denote variance and mean respectively),

$V\left( dbh \right)=c\left( E\left( dbh \right) \right)^{d}.$ (eqn A1)

Using the individual height-dbh allometry (ht denotes height)

$ht=f\left( dbh \right)^{g}, g>0$ (eqn A2)

and biomass equation (eqn 5, $\rho$ denotes wood density), we wrote

$$V\left( AGB \right)=V\left[ \frac{0.4\pi}{4}\left( dbh \right)^{2}(ht+300)\rho\right]=V\left[ \frac{0.4\pi}{4}\left( dbh \right)^{2}(f\left( dbh \right)^{g}+300)\rho\right]=V\left[ \left( c_{1}dbh^{g+2}+c_{2}dbh^{2} \right)\rho\right],$$

where $c_{1}=\frac{\pi f}{10}$ and $c_{2}=30\pi$ are constants.

Assuming statistical independence between $\rho$ and dbh and using variance formula for product of independent random variables (Goodman 1960),

$$V\left( AGB \right)=\left[ E\left( c_{1}dbh^{g+2}+c_{2}dbh^{2} \right) \right]^{2}V\left( \rho\right)+E\left( \rho^{2} \right)V\left( c_{1}dbh^{g+2}+c_{2}dbh^{2} \right)$$

$$=\left[ c_{1}E\left( dbh^{g+2} \right)+c_{2}E\left( dbh^{2} \right) \right]^{2}V\left( \rho\right)+E\left( \rho^{2} \right)\left[ c_{1}^{2}V\left( dbh^{g+2} \right)+c_{2}^{2}V\left( dbh^{2} \right)+c_{1}c_{2}cov\left( dbh^{g+2},dbh^{2} \right) \right]$$

$$=\left[ c_{1}E\left( dbh^{g+2} \right)+c_{2}E\left( dbh^{2} \right) \right]^{2}V\left( \rho\right)+E\left( \rho^{2} \right)\left[ c_{1}^{2}V\left( dbh^{g+2} \right)+c_{2}^{2}V\left( dbh^{2} \right)+c_{1}c_{2}\left( E\left( dbh^{g+4} \right)-E\left( dbh^{g+2} \right)E\left( dbh^{2} \right) \right) \right]$$

$\approx\left[ c_{1}\left( E\left( dbh \right) \right)^{g+2}+c_{2}\left( E\left( dbh \right) \right)^{2} \right]^{2}V\left( \rho\right)+E\left( \rho^{2} \right)\left[ c_{1}^{2}\left( g+2 \right)^{2}\left( E\left( dbh \right) \right)^{2\left( g+1 \right)}V\left( dbh \right)+c_{2}^{2}2E\left( dbh \right)V\left( dbh \right)+c_{1}c_{2}\left( \left( E\left( dbh \right) \right)^{g+4}-\left( E\left( dbh \right) \right)^{g+2}\left( E\left( dbh \right) \right)^{2} \right) \right]$ (eqn A3)

The last step in eqn A3 was approximated using delta method (Cramér 1946; Oehlert 1992) for the mean and variance of functions of random variables:

$$E\left( f\left( X \right) \right)\approx f\left( E\left( X \right) \right)$$

and

$$V\left( f\left( X \right) \right)\approx\left( f^{'}\left( E\left( X \right) \right) \right)^{2}V\left( X \right),$$

Assuming $\rho$ was independently and identically distributed across sites and *E*(*dbh*) was greater than one (greater than zero on log scale, see Fig. 2b). Substituting eqn A1 into eqn A3 yielded

$$V\left( AGB \right)\approx\left[ c_{1}\left( E\left( dbh \right) \right)^{g+2}+c_{2}\left( E\left( dbh \right) \right)^{2} \right]^{2}V\left( \rho\right)+E\left( \rho^{2} \right)\left[ c_{1}^{2}\left( g+2 \right)^{2}\left( E\left( dbh \right) \right)^{2\left( g+1 \right)}c\left( E\left( dbh \right) \right)^{d}+c_{2}^{2}2E\left( dbh \right)c\left( E\left( dbh \right) \right)^{d} \right]$$

$=c_{3}\left( E\left( dbh \right) \right)^{2g+4}+c_{4}\left( E\left( dbh \right) \right)^{g+4}+c_{5}\left( E\left( dbh \right) \right)^{4}+c_{6}\left( E\left( dbh \right) \right)^{2g+d+2}+c_{7}\left( E\left( dbh \right) \right)^{d+1}.$ (eqn A4)

The last term in eqn A4 was a sum of power functions of *E*(*dbh*), for which the term with the highest power would dominates the others (when *E*(*dbh*) > 1). Therefore when *d*$\leq$2, 2*g*+*d*+2 $\leq$2*g*+4 and *V*(*AGB*) behaved like (*E*(*dbh*))^2^*^g^*^+4^ for large *E*(*dbh*); when *d* > 2, 2*g*+*d*+2 $>$2*g*+4 and *V*(*AGB*) behaved like (*E*(*dbh*))^2^*^g^*^+^*^d^*^+2^ for large *E*(*dbh*).

Similarly, using delta method and under the same assumptions,

$$E\left( AGB \right)=E\left[ \left( c_{1}dbh^{g+2}+c_{2}{dbh}^{2} \right)\rho\right]=E\left( c_{1}dbh^{g+2}+c_{2}{dbh}^{2} \right)E\left( \rho\right)$$

$\approx\left[ c_{1}\left( E\left( dbh \right) \right)^{g+2}+c_{2}\left( E\left( dbh \right) \right)^{2} \right]E\left( \rho\right).$ (eqn A5)

Therefore *E*(*AGB*) behaved like $\left( E\left( dbh \right) \right)^{g+2}$ for large *E*(*dbh*).

Power exponent of Taylor's law for individual size using dbh was significantly greater than two in the Diaoluo Mountain data (*d* was 4.17 in 2010 and 4.25 in 2015). Hence the power exponent of Taylor's law for individual size using AGB was

$$\frac{d\left( \log\left( V\left( AGB \right) \right) \right)}{d(\log(E(AGB)))}=\frac{d\left( \log\left( V\left( AGB \right) \right) \right)/d(\log E(dbh))}{d(\log(E(AGB)))/d(\log E(dbh))}\approx\frac{2g+d+2}{g+2}.$$

Using the height-dbh allometric exponent g (0.51 in 2010 and 0.52 in 2015), we predicted that the power exponent of Taylor's law for individual size using AGB as 2.86 (=$\frac{2\times0.51+4.17+2}{0.51+2}$) in 2010 and 2.89 (=$\frac{2\times0.52+4.25+2}{0.52+2}$) in 2015, falling within the corresponding 95% confidence interval ((2.24, 3.15) in 2010 and (2.21, 3.20) in 2015, Table 2) estimated from data.

*Relation of power exponent of abundance-size relationship*

Suppose the abundance-size relationship holds when dbh was the size measure,

$$E\left( abd \right)=\alpha\left( E\left( dbh \right) \right)^{\beta}.$$

Using the conclusion from eqn A5, we derived

$$E\left( abd \right)\approx\alpha\left( E\left( AGB \right) \right)^{\frac{\beta}{g+2}}.$$

Using empirical estimates of $\beta$ (-1.68 in 2010 and -1.73 in 2015) and $g$ (0.51 in 2010 and 0.52 in 2015), we found that the power exponent of abundance-size relationship under AGB was -0.67 (=$\frac{-1.68}{0.51+2}$) in 2010 and -0.69 (=$\frac{-1.73}{0.52+2}$) in 2015, significantly smaller than the corresponding estimate from data (95% confidence interval is (-0.5842, -0.2052) in 2010 and (-0.6119, -0.2515) in 2015, see Table 2).

*Relation of power exponent of abundance-size variance relationship*

Suppose the abundance-size variance relationship holds when dbh was the size measure,

$$V\left( abd \right)=\gamma\left( V\left( dbh \right) \right)^{\eta}.$$

Using eqns A1 and A4,

$$V\left( abd \right)\approx\gamma\left( c\left( E\left( dbh \right) \right)^{d} \right)^{\eta}\approx c_{8}\left( V\left( AGB \right) \right)^{\frac{d\eta}{2g+d+2}}.$$

Using empirical estimates of $d$ (4.17 in 2010 and 4.25 in 2015), $\eta$ (-0.77 in 2010 and -0.70 in 2015), and $g$ (0.51 in 2010 and 0.52 in 2015), we found that the power exponent of abundance-size variance relationship under AGB was -0.45 (=$\frac{4.17\times(-0.77)}{2\times0.51+4.17+2}$) and significantly smaller than the corresponding estimate in 2010 (95% confidence interval is (-0.4219, -0.1834), see Table 2), and was ‑0.41 (=$\frac{4.25\times(-0.70)}{2\times0.52+4.25+2}$) and not different from the empirical estimate in 2015 (95% confidence interval is (-0.4281, -0.1581), see Table 2).

Overall, we derived the analytic relation of power exponent of each scaling relationship (Taylor's law for individual size, abundance-size relationship, and abundance-size variance relationship) under different size measures (AGB and dbh). Predictions using these analytic formulas yielded reasonable estimate of the power exponent for Taylor's law for individual size, but not for abundance-size relationship or abundance-size variance relationship. This finding suggested that power law as a first approximation of height-dbh allometry connected successfully the scaling exponents of Taylor's law for individual size under different measures, however it may be too crude to produce reasonable approximations of the scaling exponents of abundance-size relationship and abundance-size variance relationship under different measures.
