# Supplementary material for: Individual size variation reduces spatial variation in abundance of tree community assemblage, not of tree populations
Source: Ecol Evol. 2017 Nov 9;7(24):10815–28. doi: 10.1002/ece3.3594 (PMC5743614; doi:10.1002/ece3.3594)
Supplement: Supplementary file 14 [file ECE3-7-10815-s014.docx]

Table S1. Tree sample data from Diaoluo Mountain tropical forest in 2010 and 2015 separately. Each row records one individual with its diameter at breast height (cm), height (m), wood density (g/cm3), and aboveground biomass (g) (calculated from eqn 5). Full data are available at Dryad (doi:10.5061/dryad.87n81)
